# Improving musculoskeletal care with AI enhanced triage through data driven screening of referral letters

**DOI:** 10.1038/s41746-025-01495-4

**Published:** 2025-02-14

**Authors:** Tjardo Daniël Maarseveen, Herman Kasper Glas, Josien Veris-van Dieren, Erik van den Akker, Rachel Knevel

**Affiliations:** 1 Department of Rheumatology, Leiden University Medical Center, Leiden, Zuid-Holland the Netherlands; 2 Rheumatology outpatient clinics, Reumazorg Zuid West Nederland, Goes, Zeeland the Netherlands; 3 Leiden Computational Biology Centre, Leiden University Medical Center, Leiden, Zuid-Holland the Netherlands; 4 Delft Bioinformatics Lab, Delft University of Technology, Delft, Zuid-Holland the Netherlands; 5 Rheumatology, Newcastle University Translational and Clinical Research Institute, Newcastle upon Tyne, UK

**Keywords:** Signs and symptoms, Health care, Diagnosis, Health care economics

## Abstract

Musculoskeletal complaints account for 30% of GP consultations, with many referred to rheumatology clinics via letters. This study developed a Machine Learning (ML) pipeline to prioritize referrals by identifying rheumatoid arthritis (RA), osteoarthritis, fibromyalgia, and patients requiring long-term care. Using 8044 referral letters from 5728 patients across 12 clinics, we trained and validated ML models in two large centers and tested their generalizability in the remaining ten. The models were robust, with RA achieving an AUC-ROC of 0.78 (CI: 0.74–0.83), osteoarthritis 0.71 (CI: 0.67–0.74), fibromyalgia 0.81 (CI: 0.77–0.85), and chronic follow-up 0.63 (CI: 0.61–0.66). The RA-classifier outperformed manual referral systems, as it prioritised RA over non-RA cases (*P* < *0.001*), while the manual referral system could not differentiate between the two. The other classifiers showed similar prioritisation improvements, highlighting the potential to enhance care efficiency, reduce clinician workload, and facilitate earlier specialized care. Future work will focus on building clinical decision-support tools.

## Introduction

It is estimated that roughly 30% of visits to the general practitioner (GP) concern patients with musculoskeletal (MSK) complaints^1^. While early treatment is crucial for good outcomes, patients often face a complicated path to diagnosis and care. This is partly because initial treatment may not align with their specific condition, as different MSK issues require care from different specialists. For example, rheumatologists specialize in treating rheumatoid arthritis (RA), an autoimmune disease that causes joint swelling, tenderness, and can restrict physical activity. For RA patients, early treatment by a rheumatologist is critical to prevent irreversible joint damage.

Many patients with conditions like osteoarthritis (joint cartilage deterioration) and fibromyalgia (widespread body pain) are mistakenly referred to rheumatologists because their symptoms resemble RA^2^. However, these patients need physiotherapy or occupational therapy instead^3^. Such misreferrals burden the healthcare system and extend wait times for the patients with inflammatory arthritis unnecessarily. To reduce harm it is therefore essential to optimally triage patients, ideally without putting additional burden on the clinician as the rheumatology workforce is expected to face a deficit in Europe and the US^4,5^.

In emergency medicine, systems that enhance the triaging of patients (by predicting mortality or the need for hospitalisation or critical care) are already deployed. Moreover, these methods have shown to improve the patient triaging^6,7^. However, these prioritisation scoring systems cannot so easily be transferred to the elective care setting of the rheumatology outpatient clinic as they use patients’ vital parameters as the input variables^8^.

In many countries a GP will see the patient first and refer them to secondary care in case of diagnostic uncertainty, when specialised treatment is needed or upon requests of the patients^9^. Referral is done through written communication (the referral letter) where a GP typically includes information about the patient’s health, symptoms and lab values. Suspicions for musculoskeletal complaints will often instigate the GP to write a referral letter to a rheumatologist. Currently, RA patients wait an average of four weeks to see a rheumatologist after receiving a referral^10^. However, the total time from symptom onset to seeing a specialist averages 24 weeks, whereas treatment should ideally begin within 6 weeks of symptom onset^11^.

Referral letters provide a unique opportunity to intervene early in the care pathway. However, their use in research has been limited due to their unstructured nature and the varying writing styles of GPs^12^. With advancements in Machine Learning (ML) it is now feasible to process format free text automatically. In particular, Natural Language Processing (NLP) and large language models (LLMs) show great potential for analyzing unstructured text^13,14^. Unlike traditional statistical methods, these techniques can handle larger and more complex datasets, making them more adequate for this purpose^15^.

Despite the healthcare sector’s growing embrace of artificial intelligence, clinical adoption of Natural Language Processing is progressing slowly, even in the face of compelling research results^13^. Previous studies have explored NLP for medical triage - combining referral letters with blood tests to identify inflammatory diseases^16,17^, or with questionnaires to guide treatment for back pain^15^. However, these approaches lacked external validation, highlighting that NLP applications in healthcare remain nascent.

Building on these successes, we aim to improve triage for musculoskeletal complaints by prioritizing urgent cases and directing non-inflammatory cases to appropriate care pathways. To achieve this, we propose using NLP and ML techniques to identify :

I) patients who will likely be diagnosed with RA,

II) patients who will likely be diagnosed with osteoarthritis,

III) patients who will likely be diagnosed with fibromyalgia,

IV) patients who will likely remain under care of a rheumatologist for > 3 months (i.e. chronic)

The first classification tasks concern patients in need of different types of care. The latter is informative as chronic follow-up suggests a clear need of rheumatology care.

## Results

**Fig. 1:**
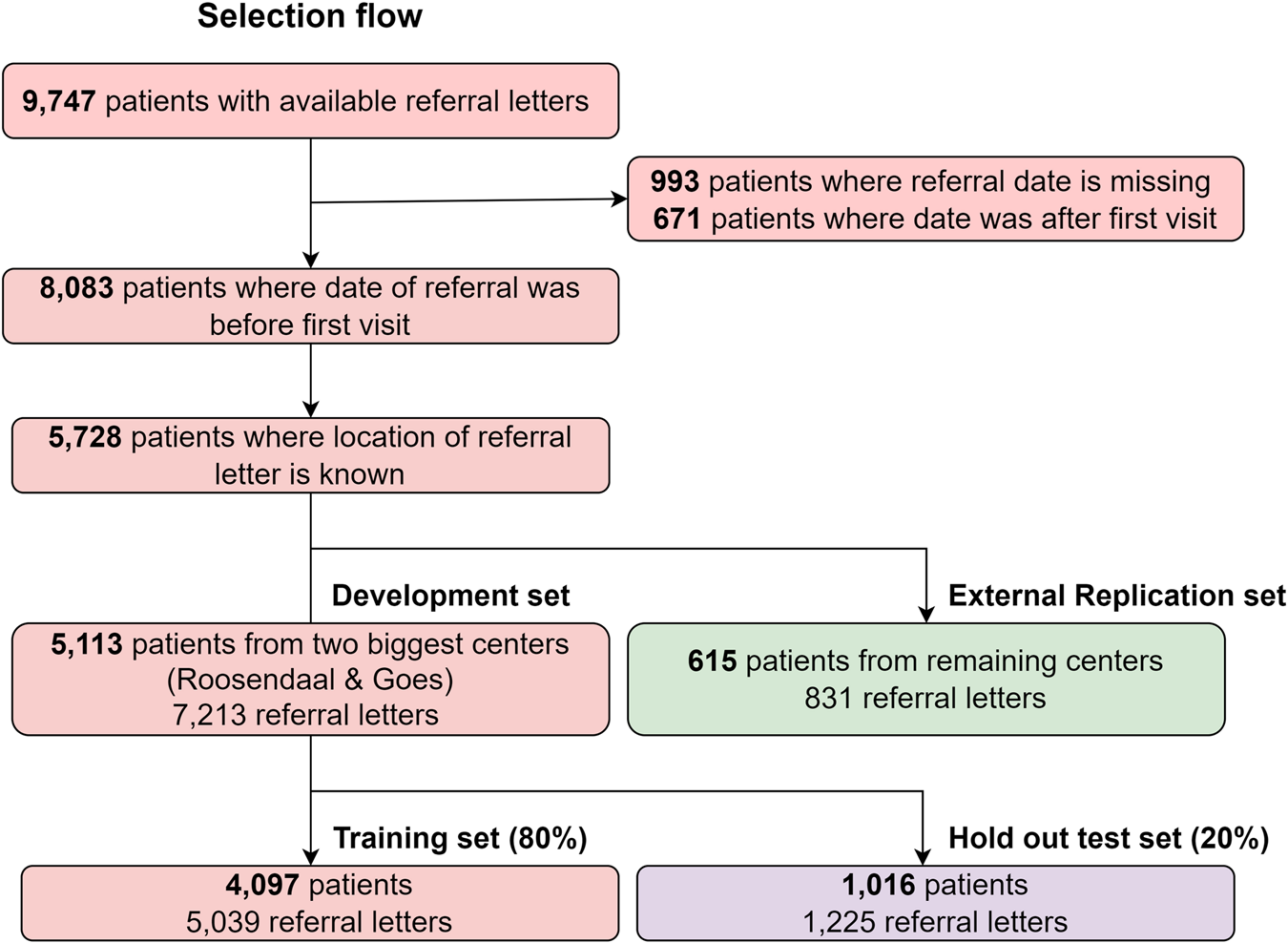
Selection flow of patients and corresponding number of (valid) referral letters. Our selection includes all referral letters from the Reumazorg ZWN initiative that were issued prior to a patient’s first visit to a rheumatologist and for which the hospital location could be determined. Two centers (Roosendaal and Goes) were chosen for the development dataset, while the remaining centers were allocated to the external replication dataset. The development dataset was further divided into two subsets: a training set and a hold-out test set.

Between 2015 and 2022, 8044 GP referral letters were sent to 12 different centers. The two largest centers (Roosendaal & Goes) were selected to develop the pipeline, using 80% of the data to train the classifier and reserving 20% as a holdout set to fairly estimate model performance. Additionally, we conducted an extra validation step to assess the classifier’s generalizability using data from the remaining ten hospitals, referred to as the ‘replication set’. A flow diagram for the GP letters and corresponding patients is shown in Fig. 1.

The training set featured 5039 valid referral letters and was used for fine tuning (Supplementary Figure 1). Of those 422 were diagnosed with RA, 1140 with osteoarthritis, 296 with fibromyalgia (Supplementary Table 1). We had 2790 referral letters that met our to-be-predicted outcome ‘chronic’ as they included patients that remained under rheumatology care for more than 3 months. The median wait time for a patient between referral and first appointment was 8 days, and median follow up of 92 days.

The hold-out validation set comprised 1225 letters, including 100 RA-, 262 osteoarthritis-, 79 fibromyalgia- and 669 chronic-follow-up cases respectively. The wait time for the validation set was 7 days, and median follow-up 86 days. The replication set featured 831 letters and comprised: 90 RA-, 218 osteoarthritis-, 37 fibromyalgia- and 448 chronic follow-up cases. The median wait time and follow up time was considerably longer in the replication set: 16 days, and 129 days follow up.

### Rheumatoid arthritis

**Fig. 2:**
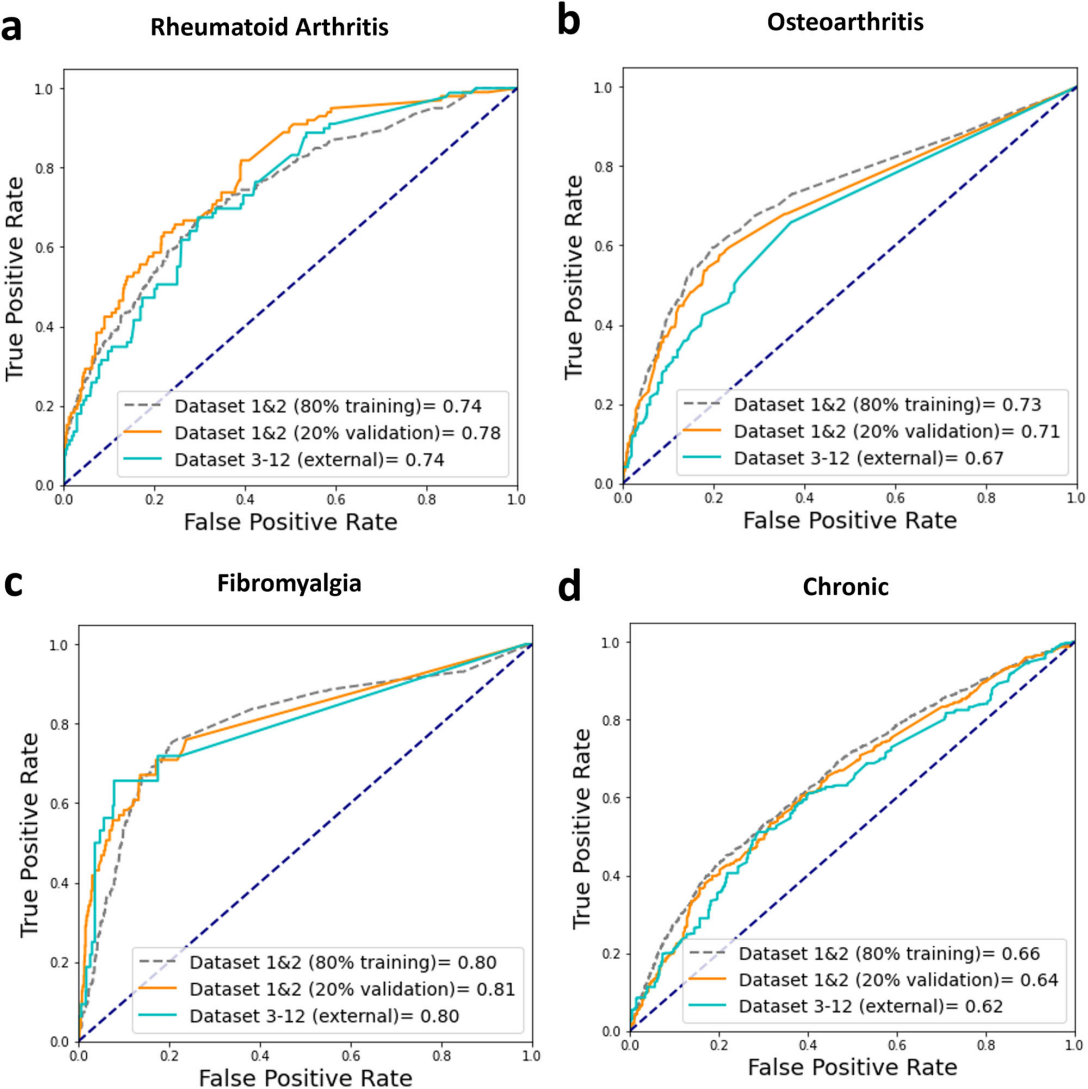
Overview of diagnostic ability for the detection of rheumatoid arthritis, osteoarthritis, fibromyalgia and patients in need of chronic follow up. The area under the curve indicates the predictive ability of the model for (**a**) Rheumatoid arthritis (**b**) Osteoarthritis (**c**) Fibromyalgia and (**d**) patients who remain under care of a rheumatologist for > 3 months (i.e., chronic). We illustrate the performance in the train (=grey), validation (=orange) and the replication set (=cyan) against the benchmark of random assignment (=blue dashed line).

**Fig. 3:**
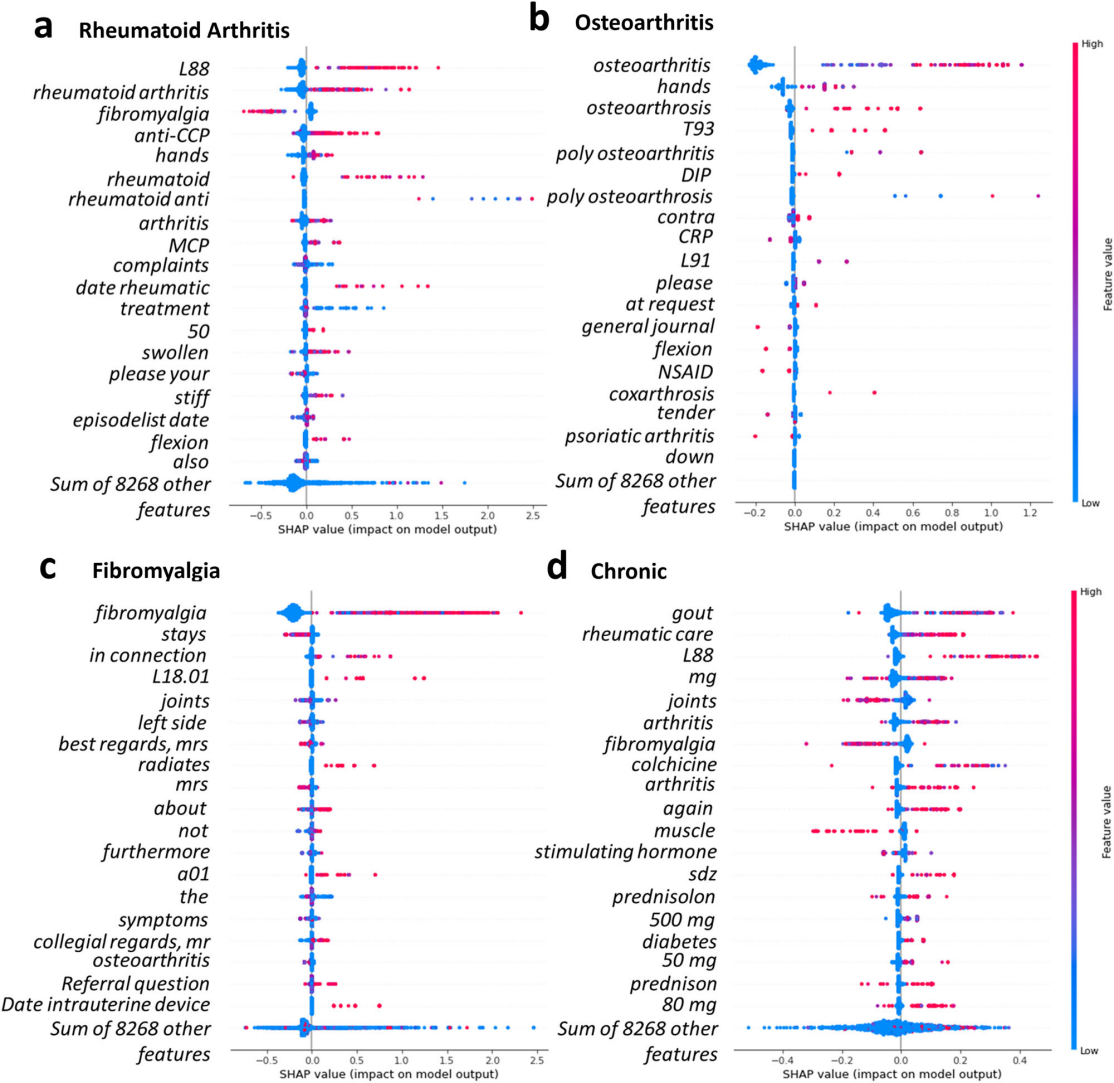
SHAP feature importance plot for the detection of rheumatoid arthritis, osteoarthritis, fibromyalgia and patients in need of chronic follow up. SHAP shows the impact and direction of the most informative words in the referral letter per patient (=dot) for the four different classification tasks: (**a**) Rheumatoid arthritis (**b**) Osteoarthritis (**c**) Fibromyalgia d patients who remain under care of a rheumatologist for > 3 months. If the words are frequently occurring for that patient its coloured pink, if it is absent its coloured blue.

The model for the RA reached an AUC-ROC of 0.78 (CI:0.74–0.83) and an AUC-PRC of 0.31 (CI:0.21–0.42) in the validation set (Fig. 2, Supplementary Figure 2). The most important keywords for RA prediction were specified location in hands or the MCP-joint (metacarpophalangeal), as well as the International Classification of Primary Care code (ICPC) for rheumatic musculoskeletal complaints (L88) (Fig. 3). Furthermore, the anatomical term flexion was also predictive, as were mentions of stiffness or swelling. Mentioning of fibromyalgia was negatively associated with having the diagnosis. The RA model showed a decent calibration, achieving a Brier score of 0.06 (Supplementary Figure 3).

### Prediction of the non-autoimmune diseases fibromyalgia and osteoarthritis

The trained models for the non-autoimmune diagnoses yielded similar AUCs in the holdout validation set: osteoarthritis, (AUC-ROC = 0.71 (CI: 0.67–0.74), AUC-PRC = 0.44 (CI: 0.40–0.52)) and fibromyalgia (AUC-ROC = 0.81 (CI: 0.77–0.85), AUC-PRC = 0.33 (CI: 0.25–0.48)). For osteoarthritis, the mentioning of hands, DIP-joint (distal interphalangeal joint) and ICPC codes for ‘other osteoarthritis’ (L91) and vitamin deficiency (T93) were positively associated, whereas mention of psoriatic arthritis and flexion were negatively associated with the outcome. When a referral letter denotes a patient’s request to be referred (e.g., ‘at request’) it was also positively associated with osteoarthritis. The important keywords for fibromyalgia included the disease-specific ICPC code (L18.01), ICPC code for generalised pain (A01), or mention of radiating pain. The mention of joints, or symptoms were negatively associated with the outcome. Notably, polite & formal greetings like ‘collegial regards, mrs’ and ‘best regards, mrs’ were also present in the top 20 most discriminatory terms.

### Prediction of patients with long term follow-up (‘chronic’)

The model for chronic patients performed less well, as we acquired a relatively low AUC-ROC of 0.63 (CI: 0.61–0.66). The AUC-PRC was 0.65 (CI: 0.61–0.68), but it is important to note that most cases fall into the ‘chronic’ category which may inflate the AUC-PRC. The keywords for the patient identification were gout, L88, rheumatic care and medication names such as colchicine, prednisolone, or specific dosages like 500 mg or 80 mg. Mentioning fibromyalgia, muscle and joints was negatively associated with being followed up by a rheumatologist.

### Performance in independent centres

To test whether the models were generalizable, we applied them to the ten additional centres and compared the AUCs across all centres (Fig. 2). Here, the RA-model yielded a similar AUC-ROC of 0.74 (CI:0.69–0.78) and AUC-PRC of 0.31 (CI: 0.21-0.38) as in the validation set. The AUCs for osteoarthritis and fibromyalgia were mostly similar, though osteoarthritis had a slightly lower AUC-ROC (0.67, CI: 0.64–0.71) the AUC-PRC was almost on equal footing with the validation set (0.45, CI: 0.40–0.51). For fibromyalgia, while the AUC-ROC was 0.80 (CI: 0.71–0.87), there was a slight decrease in the AUC-PRC (0.25, CI: 0.13–0.41). Finally, the chronic model maintained a similar performance compared to the validation set (AUC-ROC of 0.61 (CI: 0.57–0.64), AUC-PRC = 0.65 (CI: 0.61–0.69)).

**Fig. 4:**
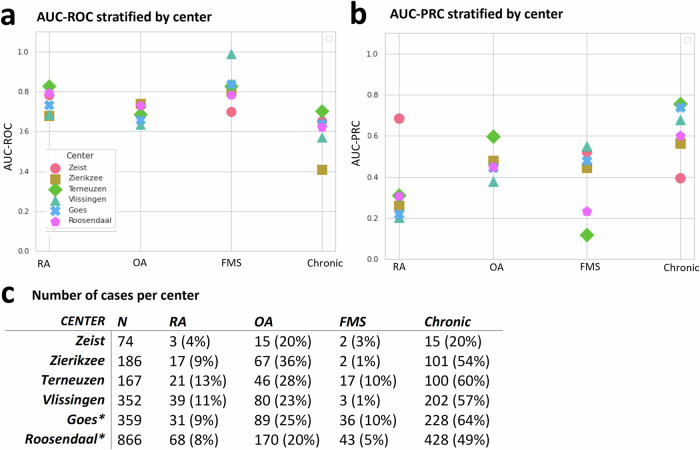
Evaluation of different classification tasks stratified per centre (only featuring those with more than 50 patients). Results showing the performance for the classification of Rheumatoid arthritis (RA), Osteoarthritis (OA), Fibromyalgia and patients who remain under rheumatologist care for more than three months (chronic), across different centers. Here, each center is represented by a unique icon, showing the (**a**) AUC-ROC performance per classifier, (**b**) the AUC-PRC and a (**c**) table detailing the case prevalence per center for reference. Where * = the performance in Goes en Roosendaal was assessed on the hold-out set that constituted 20% of data (1225 patients).

Looking at the centre-specific performance (only showing centres with at least 50 referral letters), we found that the RA and osteoarthritis models yielded stable classifiers (Fig. 4). When we adjusted for age and sex (Supplementary Figure 4), we noted minor variations in performance. The case prevalence also differed among these groups (Supplementary Table 2 & 3). The fibromyalgia- and chronic model were less stable, showing fluctuating AUC-ROCs between 0.70–1.00 and 0.40–0.70 respectively. Additionally, we notice a difference in case prevalence between the centres, with some centres reporting less than 10 cases.

### Defining a binary classifier

For RA we defined a cut-off at 0.08 based on the discrimination in the validation data. This corresponded to a sensitivity of 0.71 and NPV of 0.67 (Supplementary Figure 5). This cut-off captures two third of all RA cases while excluding two third of all non-RA cases at the same time. When we applied this cut-off to the external data, we found stable results with similar sensitivity and NPV as in the training data (Supplementary Table 4). For osteoarthritis, we set a cut-off at 0.50 (Supplementary Table 5) to capture a population where roughly two thirds (precision=0.63) end up getting the osteoarthritis diagnosis with a specificity of 0.95. For fibromyalgia we could not define a meaningful binary cut-off with high precision. For chronic conditions, we set a binary threshold at 0.55. This threshold included two-thirds of chronic patients while excluding half of the non-chronic cases (Supplementary Table 6).

### Examining models’ dependency on GPs assessment

The assessment of the GPs reason for referrals showed that 62% of the RA cases were not suspected by the GP and in 58% of suspected RA this was incorrect. These numbers were 54% missed and 33% incorrect for osteoarthritis, 44% and 38% for fibromyalgia and 31% and 26% for chronic follow-up.

**Fig. 5:**
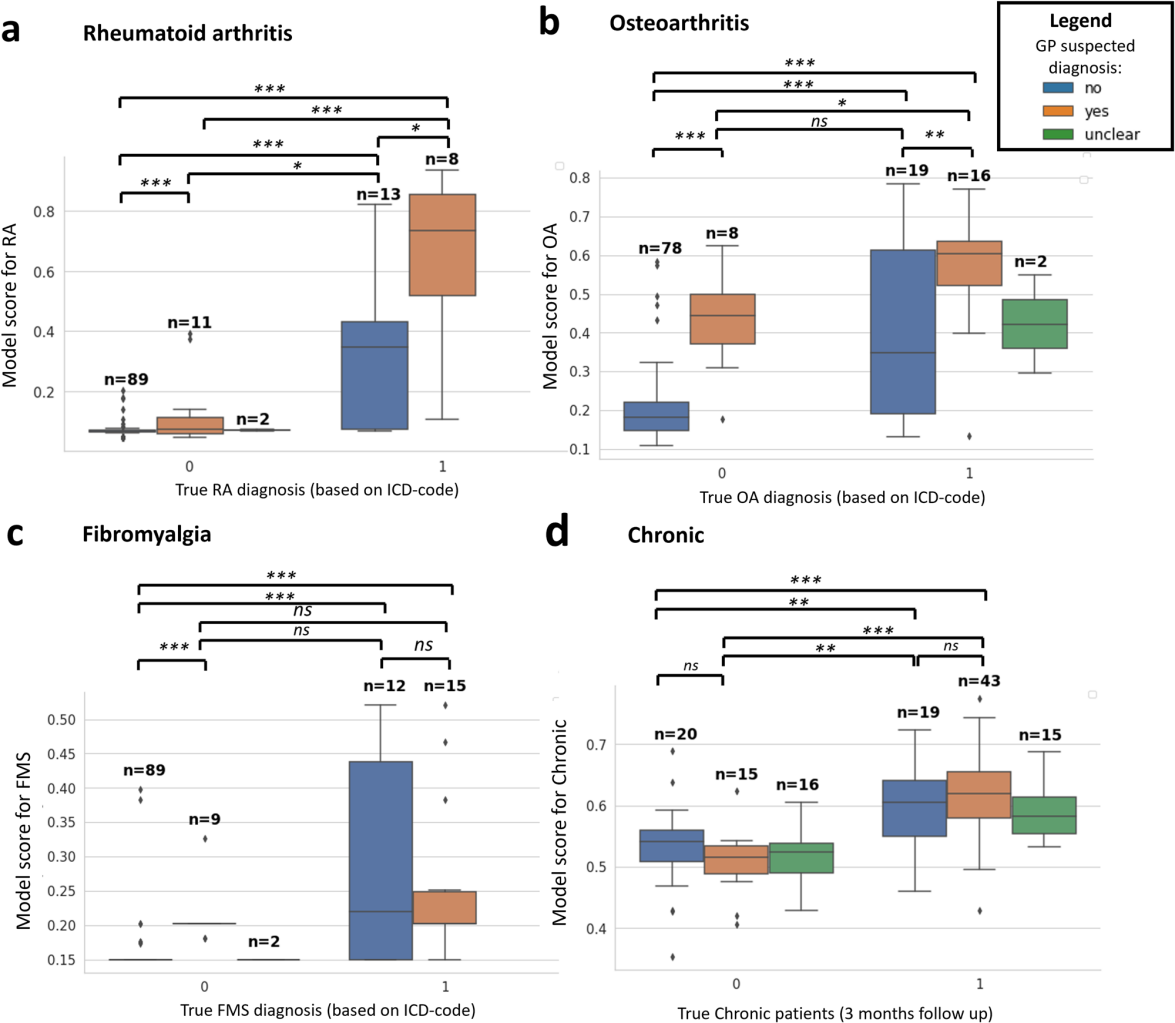
Impact of model scores (estimated probabilities) when GP mentions condition in the referral letter. **a** Boxplot showing the model score for rheumatoid arthritis (RA) compared to the true diagnosis, stratified by the GP’s suspicions on the patients’ condition. **b** Model scores for osteoarthritis. **c** Model scores for fibromyalgia. **d** Model scores for patients who remain under care of a rheumatologist for > 3 months (i.e. chronic). Where *= *p* < 0.05, **= *p* < 0.01, ***= *p* < 0.001 according to the Student’s *t*-test.

The probabilities of our prediction models were significantly higher for those with the disease versus those without, also when the GP did not explicitly suspect the disease. This shows that our model does not solely rely on the GPs reason for referral. Though, the confidence of the model did increase when GP mentions the disease (Fig. 5).

Markedly, the RA-model recognizes non-cases even in situations where the GP suspects RA as the confidence remains relatively low. There was a significant difference between the models scores of non-cases vs cases despite GP mentioning RA in both situations (*P* < *0.001)*. For osteoarthritis and fibromyalgia, the model had more difficulty identifying non-cases when the GP suggested otherwise. The chronic model identified non-cases even when the GP suspected a patient needed rheumatological care.

Next, we examined the differences in cases identified by the ML method and the general practitioner (Supplementary Figure 6), noting that these do not reflect real-world prevalences. In this annotation set, ML demonstrates higher sensitivity than GP for detecting RA (with AUC-ROC scores of 0.75 vs. 0.63), osteoarthritis (0.87 vs. 0.75), and fibromyalgia (0.90 vs. 0.77). For RA the ML-model was also more precise than the GP (AUC-PRC of 0.53 vs 0.48). Our models were less precise compared to GP suggestions for both osteoarthritis (0.55 vs. 0.68) and fibromyalgia (0.54 vs. 0.66). Furthermore, the ML was less successful in identifying the chronic patients compared to the GP: reaching an AUC-ROC of 0.57 vs 0.85 and AUC-PRC of 0.68 vs 0.91 for ML and GP respectively.

### Waiting time prioritisation based on referral letter

**Fig. 6:**
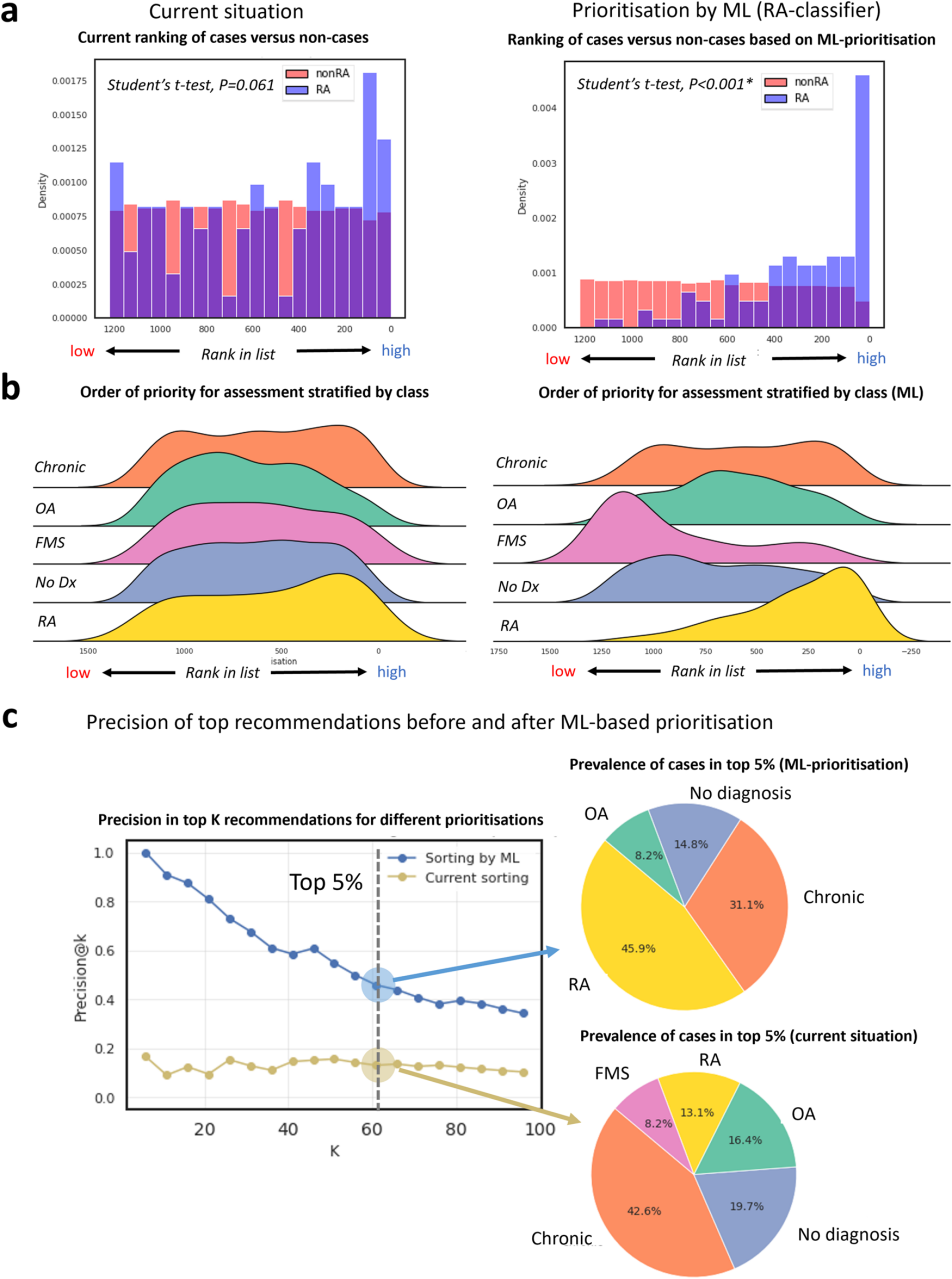
Comparison between the current visit order of patients vs prioritisation using the rheumatoid arthritis (RA)-classifier. **a** the distribution of cases (blue) and non cases (red) across the present visit order (ranked by wait time) vs machine learning (ML) based prioritisation by the RA-classifier. Here, we quantified the discernment in appointment order between cases and non-cases based on the Student’s *t*-test. **b** the distribution of each class before and after ML prioritisation. **c** the precision of the top recommendations accompanied by the class prevalence for the top 5% letters (*n* = 61) according to both strategies.

Finally, we assessed whether the ML-methods could expedite the assessment of RA-cases by comparing the wait time between RA-cases versus non-RA cases. In the real life situation, there was no significant difference between the wait time of RA and non-RA cases (*P* = *0.061)*. In contrast, the RA-classifier ranked most of the RA-cases to the front of the line (*P* < *0.001;* Fig. 6a, b). This ML-based prioritisation had fewer false positives and achieved greater precision than the current appointment ordering (Fig. 6c).

Zooming in at the top 5%, we see that the top rows are filled with the majority of RA-cases (45.9% with the ML model versus 13.1% in reality; Fig. 6). Interestingly, there were also some non-cases with high model scores. When we studied these we found that these letters often contained words like: ‘RA’ or ‘L88’ (Supplementary Figure 7). Another interesting detail is that the RA-classifier appears to put the fibromyalgia letters at the bottom of the list (Fig. 6b). Notably, the osteoarthritis, fibromyalgia and chronic models also meet their prioritisation objective (Supplementary Figures 8 & 9).

## Discussion

In this study, we developed an NLP pipeline to automate referral prioritization for patients with musculoskeletal disorders based on their GP letters. Using only the contents of the letters, we accurately predicted patients with RA, osteoarthritis, and fibromyalgia prior to their first secondary care visit at the rheumatology outpatient clinic. We validated the ML-models across multiple hospitals, showing the models for individual diagnosis perform well regardless of each hospital-specific structure. Only the heterogenous group of patients with chronic follow-up proved to be challenging to predict. Nonetheless, all the ML models improved the referral order compared to the manual referral system, placing the high-risk patients to the front of the line.

We believe this AI-assisted patient triage shows significant promise for reducing wait times and alleviating clinician workload. In many Western countries, including the Netherlands, an aging population and fewer specialists - especially in peripheral areas - exacerbate delays^4,5^. Additionally, many patients are referred to incorrect care pathways, further delaying access to rheumatologists. As a result, patients with MSK symptoms often feel trapped in a “limbo” within the healthcare system^18^.

Healthcare providers could implement this pipeline^19^ to improve the prioritisation of referral letters based on estimated urgency. Specifically, the RA model could be used to create a “fast-track” pathway to the specialist for early intervention with immunosuppressants. Meanwhile, non-autoimmune conditions like fibromyalgia and osteoarthritis could be directed to alternative care pathways, such as occupational- or physiotherapy.

Aside from secondary care, this pipeline also shows promise for primary care. The model’s ability to predict the correct diagnosis, even when the GP did not explicitly mention it in the referral reason, demonstrates its value as a supportive tool for GPs. Based on the description of the patient’s condition it could help GPs decide when to order additional tests or prioritize referrals. However, future research should evaluate the model’s performance on all patients considered for referral, not just those actually referred. Since we selected cases based on final diagnoses to ensure adequate representation, we couldn’t assess GPs’ overall predictive accuracy.

A key strength of our study is its use of referral letters, which are routinely collected in many healthcare systems as part of standard care^9^. These letters offer critical insights into a patient’s condition at early stages of the disease, enabling timely detection and intervention. This is particularly crucial for RA, where early treatment during the “window of opportunity” can significantly improve patient outcomes^20,21^.

Additionally, our models provide probabilities for multiple diseases, offering flexibility to align with the specific triage priorities of different clinics. The RA model, with strong calibration (Brier score = 0.06), is especially interpretable, as its thresholds closely reflect true RA prevalence. While these models are not designed to replace a specialist’s diagnosis, they serve as valuable tools to assist clinicians by guiding diagnostic and therapeutic decisions based on their outputs.

Natural language processing and large language models remain underutilized in the field of rheumatology, despite their potential to analyse the vast amounts of clinical text data^13,22^. While Krusche et al. applied LLMs for triage^14^, they didn’t examine the value of referral letters. Wider adoption of LLMs may be hindered by concerns about data privacy and lack of infrastructure for hosting^23^. That said, referral letters have been analysed with advanced NLP techniques in the past—for example, to differentiate inflammatory from non-inflammatory diseases^16,17^ or to triage patients with lower back pain^15^. However, these studies did not include external validation, limiting their generalizability.

Our project is the first to create a generalizable NLP pipeline for predicting and prioritizing diagnoses from referral letters for the rheumatology outpatient clinics. The SHAP-analysis shows that detailed information on suspected diagnoses, serology, lab results, treatments, and specific symptom descriptions, such as joint locations, are the most valuable features for classification. This aligns with the structured referral letter framework proposed by Engels et al.^24^. Nevertheless, we do want to emphasize the importance of validating and optimizing models locally to ensure optimal performance on the local referral letters^25^. In theory, our pipeline should be adaptable across languages, as it uses techniques (such as the BERT transformers) available for most languages. However, further investigation is needed to evaluate the transferability of our approach across different healthcare systems, given the possible variations in GP roles and triage protocols between countries^9^.

It is important to acknowledge that we did not investigate alternative machine learning techniques or perform sensitivity analyses in this study. While we focused on fine-tuning XGBoost due to its established effectiveness in text classification^26–28^, exploring other methods or text processing approaches, such as lemmatization or stemming, could potentially enhance performance^29^.

We aim to integrate ML classifiers into a decision support tool for triaging patients and aiding GPs in selecting appropriate referral targets. Building a decision support tool enters into the transdisciplinary field of human centred AI systems^30^, requiring collaboration with all stakeholders (patients, clinicians, GPs). Rather than optimising only on metrics like AUC-ROC or the F1-score, the focus will lie on optimising human centric outcomes (such as user experience, explainability, transparency and fairness). Ideally, the tool will include visualizations to enhance clinicians’ understanding of model reasoning—for example, highlighting sections of the narrative indicating musculoskeletal complaints (via SHAP analysis) or comparing a patient’s narrative with those of past patients to find similarities. We also propose evaluating average wait times for different patient groups and screening efficiency in a pilot study.

Identifying chronic patients—those requiring ongoing rheumatologist care—was challenging, likely due to the group’s heterogeneity. Compared to GPs, the ML model struggled more to predict patients needing follow-up care beyond three months. However, since GPs can request rheumatologists to take over care, their higher performance is somewhat expected. Additionally, our findings suggest that GPs adjust their writing style when transferring care for fibromyalgia patients. SHAP analysis revealed that GPs often use more formal and courteous language (e.g., “collegial regards” or “best regards”) in these referral letters.

Due to low disease prevalence, we used AUC-PRC alongside AUC-ROC, as AUC-ROC alone can be misleading for imbalanced datasets^31^. AUC-PRC shows how precise the selection is at specific sensitivity levels. For RA, which has a low prevalence of 8-10%, the model consistently achieved a higher AUC-PRC compared to the known prevalence, indicating that it prioritized actual cases more effectively than random chance would suggest. In contrast, the “chronic” category also achieved a higher AUC-PRC, but this was less impressive due to its higher prevalence in the different hospitals (53–55%), which inflates the AUC.

The fact that the ML models reached a good performance does not per definition translate to an improvement in the real world patient triaging, as this is always dependent on various factors other than performance. First, the binarized ML classifier approach may not reflect real-world scenarios where patients often have multiple diagnoses. Second, it is unclear how users will interact with automated triage systems or how much control they will want over them.

We acknowledge several limitations with the data. First, the performance could likely be increased if we had more data, given that GP letter quality varies due to inconsistent formatting^12,32^ it is important to have a large representative sample size. While we lacked GP-specific data, our study included referrals from ~300 practices across multiple Dutch provinces, probably capturing a decent range of letter quality.

Second, the codified diagnoses used as outcomes may be misclassified. Although the respective ICD codes generally remain consistent throughout a patient’s follow-up, we found that approximately 5% of diagnosed cases eventually receive a second codified diagnosis, suggesting either dual conditions or initial misclassification. Manual data cleaning could have improved diagnostic accuracy and model performance. Finally, our study does not significantly address diagnostic delays for rare diseases^33^. While our models enhance efficiency for common musculoskeletal complaints, training rare disease models was infeasible due to insufficient data.

In conclusion, using GPs’ referral letters only, we built AI-tools that could improve prioritisation for patients with RA, Osteoarthritis and Fibromyalgia, before they even visited a specialist. For all classification tasks, we were able to improve visit prioritisation compared to the current waiting time. This demonstrates its potential to optimise care efficiency. The use of ML based referral prioritisation could both reduce workload of clinicians as well as facilitate early dedicated care possibly in specified care pathways. Future research is required to translate the models into a complete decision support tool for clinical use.

## Methods

**Fig. 7:**
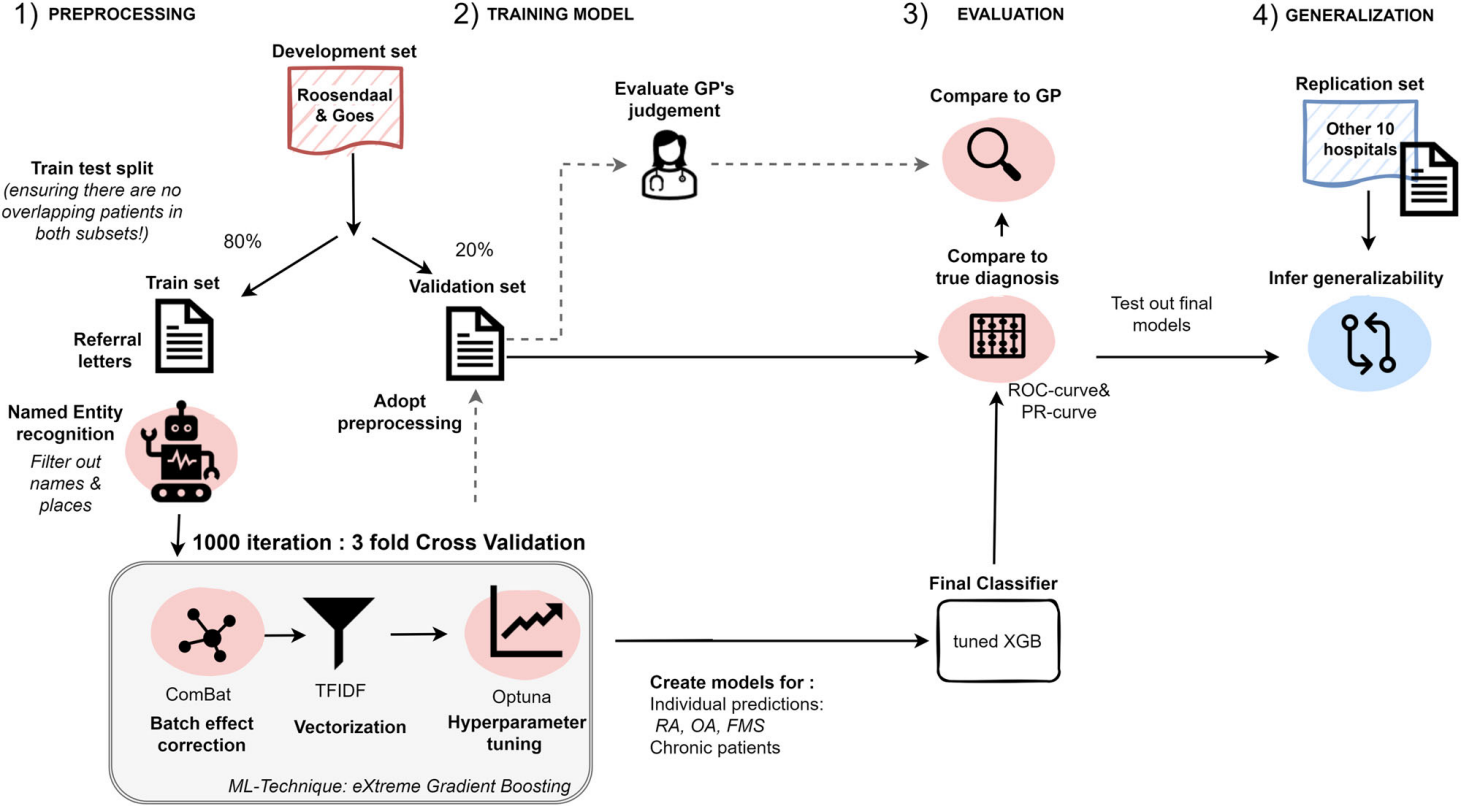
General flow showing the different steps of our study. We first divide the centers into a developmental set and a replication set, after which we start building the model on the developmental data by using a training partition to finetune the model, while testing it on the validation partition for an unbiased evaluation. Finally, we compare the model to the GP, and test for its generalizability to the other hospitals featured in the replication set.

Our dataset contained 8,044 GP referral letters, written in Dutch, from patients who visited a collective of 12 rheumatology outpatient clinics (Reumazorg ZWN) between 2015 and 2022. These letters were processed through a machine learning pipeline (Fig. 7) to predict I) RA, II) osteoarthritis, III) fibromyalgia and IV) patients who remain under the care of a rheumatologist for > 3 months. To develop the pipeline, we used data from the two largest centers, Roosendaal and Goes (*n* = 7213). Of this, 80% (*n* = 5039) was used to train a classifier, while 20% (*n* = 1225) was reserved as a validation set to fairly assess the classifier’s performance, ensuring no patient overlap between training and validation data.

To further evaluate the classifier’s generalizability, we conducted a second validation step using letters from 10 smaller centers (Zeist, Zierikzee, Terneuzen, Vlissingen, Hulst, Emmeloord, Middelburg, Lemmer, Lelystad, and Oostburg; total *n* = 831) to confirm the model wasn’t overfitting to specific characteristics of Roosendaal and Goes. Additionally, we estimated the model’s performance at individual centers with more than 50 letters.

To ensure that GP was blinded from the experts opinion, we only included letters sent prior to a first clinic visit. In accordance with the FAIR (Findable Accessible Interoperable Reusable) principles, we have made the pipeline publicly available on GitHub^19^.

We defined the diagnoses using the 10^th^ edition of the International Classification of Diseases (ICD) codes (Supplementary Table 7). As ICD based misclassification is an issue with RA, we additionally required the initiation of a disease modifying anti-rheumatic drug and at least 3 months of follow up to classify patients as a case. For each disease we trained and modelled on the training data across 1000 epochs, following a 3-fold cross-validation scheme.

### Ethical considerations

Our study is in line with the declaration of Helsinki and national guidelines, such as the COREON Code of Conduct for Health Research. Data was provided under the exception for informed consent (Article 7:458, paragraph 1, subsection b of the Dutch Civil Code) by the regional ethics committee, given that it entails a very high number of patients (15,000+ patients) and uses already available data.

The conditions for invoking this exemption were met since the data is pseudonymized and processed securely (technically), the research serves the public interest, the research cannot be conducted without the requested data. Furthermore, patients who have objected (opt-out) to the use of their data for scientific research are excluded, in accordance with Article 7:458, paragraph 1, subsection b, and paragraph 2 of the Dutch Civil Code.

### Natural language processing

We conducted a dedicated preprocessing pipeline, consisting of the following steps: 1) entity removal, 2) vectorization and 3) batch correction. Firstly, we adopted the Dutch transformer embeddings from BERTje^34^ and the sentence tagger NER-dutch^35^ to detect named entities in the referral letters. We removed personal and location names from the text because their predictive ability (if any) would be highly specific to the centre in question (e.g. name of a specialist or specific center). Specifically, we created a filter list that included all of the entities with a confidence score above 0.8 (Supplementary Figure 10) and let a rheumatologist screen through this list to define which words should be redacted.

Within each training round, we created a vocabulary of all words that were at least present in 1% in all documents from the training fold, and vectorized the text accordingly. To enhance the meaningfulness of the text we reduced the weighting of frequently occurring unimportant words (e.g. “he” or “this”) with a term frequency by inverse document frequency (TF-IDF). Finally, we used pyCombat^36^ as batch correction to deal with centre-specific language.

### Training the model

We applied the eXtreme Gradient Boosting (XGB) technique to build separate prediction models for each disease. Hyperparameter tuning was done with Bayesian optimization using a Tree-structured Parzen Estimator (Supplementary Table 8)^37^. We measured the performance in a holdout set according to the AUC-ROC (=c-statistic) and AUC-precision recall curve (AUC-PRC) (= balance between PPV and sensitivity). To identify the most important words in the referral letters, we quantified the importance with the Shapley additive explanations (SHAP) value^38^. We rendered a SHAP beeswarm plot to visualise the top 20 most relevant words.

### Replication of the model

We repeated the analysis across external datasets, to test whether the models reached a similarly high performance based on the AUC-ROC and AUC-PRC. Furthermore, we defined an optimal cut-off in the validation set, that we set out to replicate in the external set (containing the remaining ten clinical datasets).

For classification of I) RA, we aimed for a high sensitivity and negative predictive value (NPV), as type II errors would be most harmful. For classification II) osteoarthritis and III) fibromyalgia, we aimed for a high specificity and positive predictive value. Finally for classification IV) chronic patients, we aimed for high sensitivity and NPV.

### Evaluating added value of the model

To infer if the model was simply ‘extracting’ the GP’s suspicion or if it identified patterns in the text related to the disease, we studied the suspected diagnosis formulated by the GPs. A rheumatologist [RK] annotated 20 positive and 20 negative cases for each class (RA, osteoarthritis, fibromyalgia and chronic; *n* = 160 in total) in a randomised and blinded manner. Based on this annotation of the reason for referral, we could determine whether the GP had already suspected the disease or if the model identified informative structure in the narrative. For the individual’s diagnosis a simple mentioning by the GP in the reason for referral would suffice, whereas chronic was defined by the GP requesting the rheumatologist to take over the care of a patient.

Finally, we examined the differences between the current situation (based on the observed wait time) and the prioritised list (ranked on the RA-classifier). To assess the quality of prioritisation, we compared the current appointment order between RA and non RA-cases for both situations. Statistical significance was determined using Student’s *t*-test with a significance threshold of α < 0.05. In addition, we also inferred the improved efficiency of the ML-based ranking by calculating the prevalence of correct ‘recommendations’ in the top ranking letters. Here, precision was used as a metric to assess the model’s capability in minimizing false positives, as misassigning patients to incorrect care pathways is believed to impose the greatest burden on the healthcare system.

While our primary focus was to rank RA-patients at the top, given that it is the target population, we also examined the prioritisation capabilities of the other models, for a comprehensive overview.

## Supplementary information

Supplementary information

## Data Availability

The study data is available upon reasonable request.

## Code availability

The underlying code for this study is available at Github and can be accessed via this link: https://github.com/levrex/Referral_ML.
