# Supplementary material for: Improving musculoskeletal care with AI enhanced triage through data driven screening of referral letters
Source: NPJ Digit Med. 2025 Feb 14;8:98. doi: 10.1038/s41746-025-01495-4 (PMC11825706; doi:10.1038/s41746-025-01495-4)
Supplement: Supplementary file 1 — Supplementary information [file 41746_2025_1495_MOESM1_ESM.pdf]

Supplementary Information File I: additional tables

Supplementary tables from "Improving Musculoskeletal Care with AI Enhanced Triage Through Data Driven Screening of Referral Letters" by Tjardo Daniel Maarseveen, Herman Kasper Glas, Josien Veris-van Dieren, Erik van den Akker, Rachel Knevel.

Supplementary Table 1: Characteristics of the referral letter population from the training (80% of letters from Roosendaal and Goes), hold-out (20% of the letters) and replication set (letters from the remaining 10 centres).

Set	Train	Hold-out	Replication
N letters	5039	1225	831
Pat	4097	1016	615
DMARD [n(%)]	664 (16.0)	170 (16.5)	104 (16.7)
Sex, F [n(%)]	2812 (67.8)	699 (68.0)	405 (65.2)
Age (mean+-sd, yr)	54.1 (16.4)	53.4 (16.0)	54.7 (17.2)
n RA (%)	422 (8.4)	100 (8.2)	90 (10.8)
n OA (%)	1140 (22.6)	262 (21.4)	218 (26.2)
n FMS (%)	296 (5.9)	79 (6.4)	37 (4.5)
n Chronic (%)	2790 (55.4)	669 (54.6)	448 (53.9)
Wait time, days (median+-IQR)	8 (4-15)	7 (4-14)	16 (8-31)
Visits (median+-IQR)	3 (2-9)	3 (2-8)	3 (2-7)
Follow up (median+-IQR)	92 (13-784)	86 (12-693)	129 (14-567)

Where Pat, number of patients; DMARD, disease modifying anti rheumatic drug; F, Female; IQR, interquartile range; RA, rheumatoid arthritis; OA, osteoarthritis; FMS, fibromyalgia syndrome;

Supplementary Table 2: Characteristics of the referral letter population stratified on sex.

	Male	Female
N letters	2246	4849
Pat	1863	3865
DMARD [n(%)]	382 (20.3)	556 (14.2)
Sex, F [n(%)]	0 (0.0)	3916 (100.0)
Age (mean+-sd, yr)	57.0 (15.8)	52.6 (16.5)
n RA (% n)	225 (10.0)	387 (8.0)
n OA (% n)	373 (16.6)	1247 (25.7)
n FMS (% n)	11 (0.5)	401 (8.3)
n Chronic (% n)	1423 (63.4)	2484 (51.2)
Wait time, days (median+-IQR)	8 (4-16)	8 (5-17)
Visits (median+-IQR)	5 (2-11)	3 (2-7)
Follow up (median+-IQR)	266 (15-968)	43 (10-608)

Where Pat, number of patients; DMARD, disease modifying anti rheumatic drug; F, Female; IQR, interquartile range; RA, rheumatoid arthritis; OA, osteoarthritis; FMS, fibromyalgia syndrome;

Supplementary Table 3: Characteristics of the referral letter population stratified on age quartile

Age quartiles	Q1 (4 - 44)	Q2 (44 - 55)	Q3 (55 - 67)	Q4 (67 - 100)
N letters	1890	1815	1804	1586
Pat	1524	1429	1460	1315
DMARD [n(%)]	190 (12.4)	203 (13.9)	273 (18.5)	272 (20.5)
Sex, F [n(%)]	1149 (74.7)	1066 (73.2)	900 (61.1)	801 (60.4)
Age (mean+-sd, yr)	32.5 (8.2)	50.5 (3.1)	61.0 (3.4)	75.1 (5.5)
n RA (% n)	94 (5.0)	125 (6.9)	171 (9.5)	222 (14.0)
n OA (% n)	77 (4.1)	390 (21.5)	656 (36.4)	497 (31.3)
n FMS (% n)	186 (9.8)	154 (8.5)	62 (3.4)	10 (0.6)
n Chronic (% n)	906 (47.9)	903 (49.8)	992 (55.0)	1106 (69.7)
Wait time, days (median+-IQR)	8 (5-18)	9 (5-18)	8 (4-17)	7 (4-14)
Visits (median+-IQR)	2 (2-5)	3 (2-6)	3 (2-10)	6 (2-12)
Follow up (median+-IQR)	30.5 (10-378)	35.0 (10-565)	114 (14-806)	378 (21-1062)

Where Q1-Q4, first to fourth quartile respective of age (defined in the training set); Pat, number of patients; DMARD, disease modifying anti rheumatic drug; F, Female; IQR, interquartile range; RA, rheumatoid arthritis; OA, osteoarthritis; FMS, fibromyalgia syndrome.

Supplementary Table 4: Resulting confusion tables of RA classification algorithm on the validation set and test set as chosen cut-offs.

Validation set (threshold=0.08)			Test set (threshold=0.08)		
Reference	*Non-RA*	*RA*	Reference	*Non-RA*	*RA*
Non-RA	751 (TN)	375 (FP)	*Non-RA*	462(TN)	280(FP)
RA	29 (FN)	70 (TP)	*RA*	27 (FN)	62 (TP)

Metrics

Sens	0.71	*Sens*	0.70
Spec	0.67	*Spec*	0.62
PPV	0.16	*PPV*	0.18
NPV	0.99	*NPV*	0.94
Accuracy	0.67	*Accuracy*	0.63

TN = True negative, FP = False positive, FN = False negative, TP = True positive, Sens = Sensitivity, Spec = Specificity, PPV = Positive Predictive value, NPV = Negative Predictive Value, Acc = Accuracy,

Supplementary Table 5: Resulting confusion tables of osteoarthritis classification algorithm on the validation set and replication set as chosen cut-offs.

Validation set (threshold=0.50)			Test set (threshold=0.50)		
Reference	*Non-OA*	*OA*	Reference	*Non-OA*	*OA*
Non-OA	937 (TN)	29 (FP)	*Non-OA*	595 (TN)	19 (FP)
OA	210 (FN)	49 (TP)	*OA*	188 (FN)	29 (TP)

Metrics

Sens	0.19	*Sens*	0.13
Spec	0.99	*Spec*	0.97
PPV	0.63	*PPV*	0.60
NPV	0.82	*NPV*	0.76
Accuracy	0.81	*Accuracy*	0.75

TN = True negative, FP = False positive, FN = False negative, TP = True positive, Sens = Sensitivity, Spec = Specificity, PPV = Positive Predictive value, NPV = Negative Predictive Value, Acc = Accuracy,

Supplementary Table 6: Resulting confusion tables of the chronic classification algorithm on the validation set and replication set as chosen cut-offs.

Validation set (threshold=0.55)			Test set (threshold=0.55)		
Reference	*No chronic*	*chronic*	Reference	*No chronic*	*chronic*
No chronic	283 (TN)	286 (FP)	*No chronic*	186 (TN)	202 (FP)
chronic	212 (FN)	444 (TP)	*chronic*	150 (FN)	293 (TP)

Metrics

Sens	0.68	*Sens*	0.66
Spec	0.50	*Spec*	0.48
PPV	0.61	*PPV*	0.59
NPV	0.57	*NPV*	0.55
Accuracy	0.59	*Accuracy*	0.58

TN = True negative, FP = False positive, FN = False negative, TP = True positive, Sens = Sensitivity, Spec = Specificity, PPV = Positive Predictive value, NPV = Negative Predictive Value, Acc = Accuracy,

Supplementary Table 7: ICD disease codes used in this study to define disease populations

Disease	International Classification of Diseases (ICD)
Rheumatoid arthritis	'M06.99', 'M06.09', 'M05.99', 'M06.90', 'M05.29', 'M05.19+'
Fibromyalgia	'M79.79’
Osteoarthritis	'M19.94', 'M19.99', 'M17.9', 'M16.9', 'M19.91', 'M19.97', 'M19.98', 'M19.93', 'M15.9', 'M15.1', 'M15.2', 'M15.4', 'M15.9', 'M16.9', 'M17.9', 'M19.02', 'M19.03', 'M19.04', 'M19.09', 'M19.91', 'M19.93', 'M19.94', 'M19.97', 'M19.98', 'M19.99'

Supplementary Table 8 : Search space used for the Bayesian optimization of the hyperparameters for the different eXtreme Gradient Boosting models.

Parameter	Search space (range)
Objective	binary:logistic
Num class	1
Eval metric	logloss
Booster	gbtree
Lambda	loguniform(1e-8, 1.0)
Alpha	loguniform(1e-8, 1.0)
Learning rate	float(0.01, 0.3)
Subsample	float(0.5, 1.0)
Colsample bytree	float(0.5, 1.0)
Max depth	int(1, 9)
Eta	loguniform(1e-8, 1.0)
Gamma	loguniform(1e-8, 1.0)
Grow policy	["depthwise", "lossguide"]

Supplementary Information File II: additional figures

Supplementary figures from “Improving Musculoskeletal Care with AI Enhanced Triage Through Data Driven Screening of Referral Letters” by Tjardo Daniel Maarseveen, Herman Kasper Glas, Josien Veris-van Dieren, Erik van den Akker, Rachel Knevel.

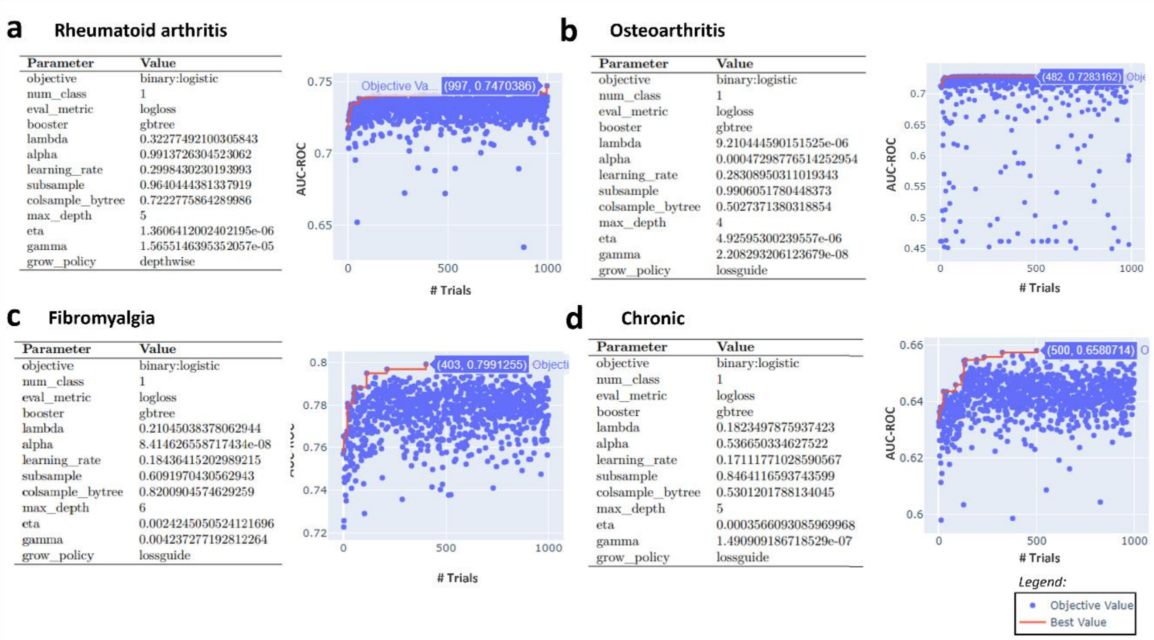

Supplementary Figure 1: The resulting parameters for the different tasks according to their optimization history across 1000 trials of hyperparameter tuning. *We show the optimization curves for the four classification tasks:* ***a*** *Rheumatoid arthritis* ***b*** *Osteoarthritis* ***c*** *Fibromyalgia* ***d*** *patients who remain under care of a rheumatologist for >3 months. Here, the best iteration is marked on the optimization curve together with the corresponding training AUC-ROC.*

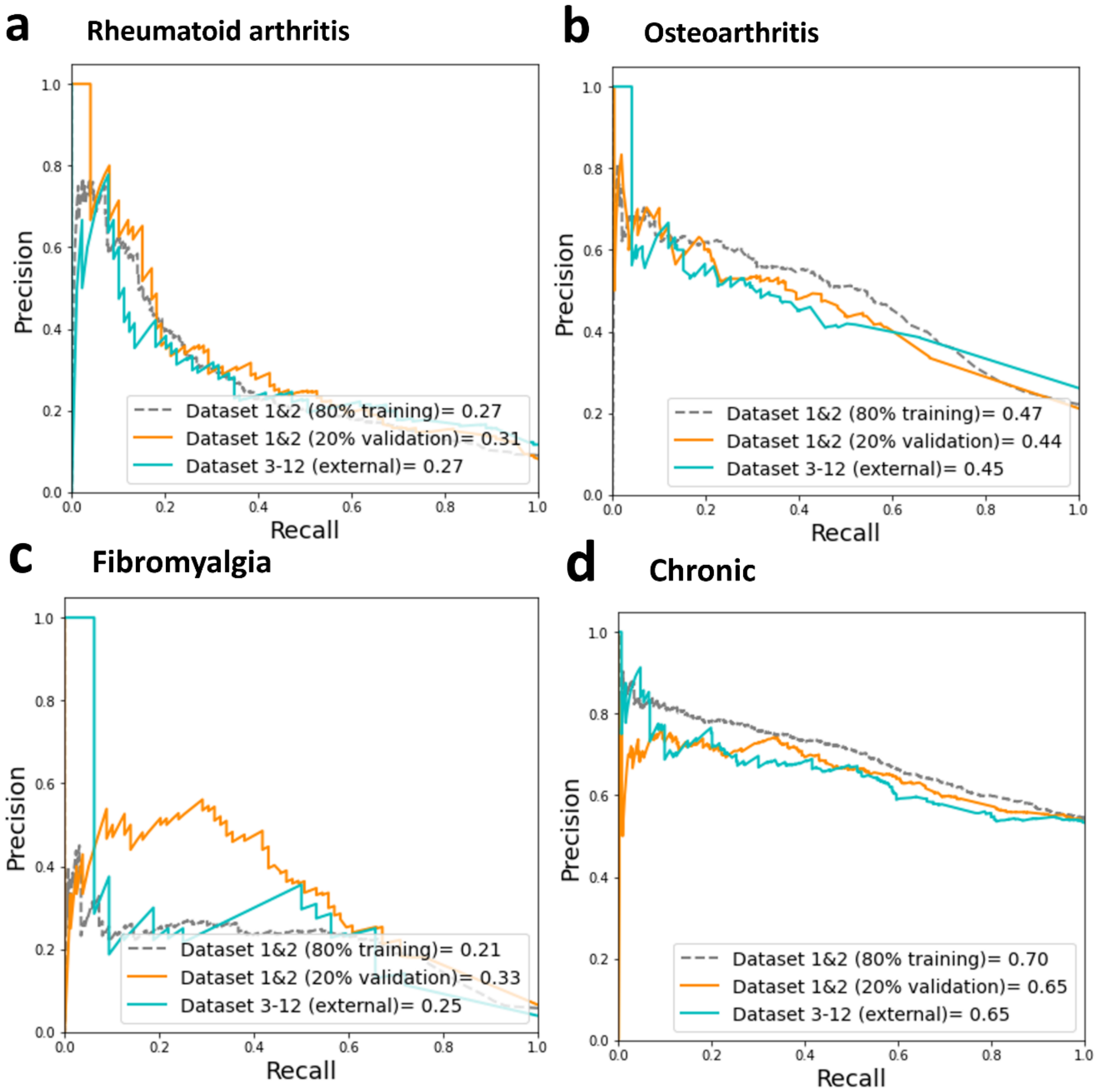

Supplementary Figure 2: Overview of precision recall curves for the classification of rheumatoid arthritis, osteoarthritis, fibromyalgia and patients in need of chronic follow up. *We show the performance for the four classification tasks:* ***a*** *Rheumatoid arthritis* ***b*** *Osteoarthritis* ***c*** *Fibromyalgia* ***d*** *patients who remain under care of a rheumatologist for >3 months. Where the area under the curve indicates the balance between the precision and sensitivity (recall) of the model in the train- (=grey), validation- (=orange) and replication set (=cyan) respectively.*

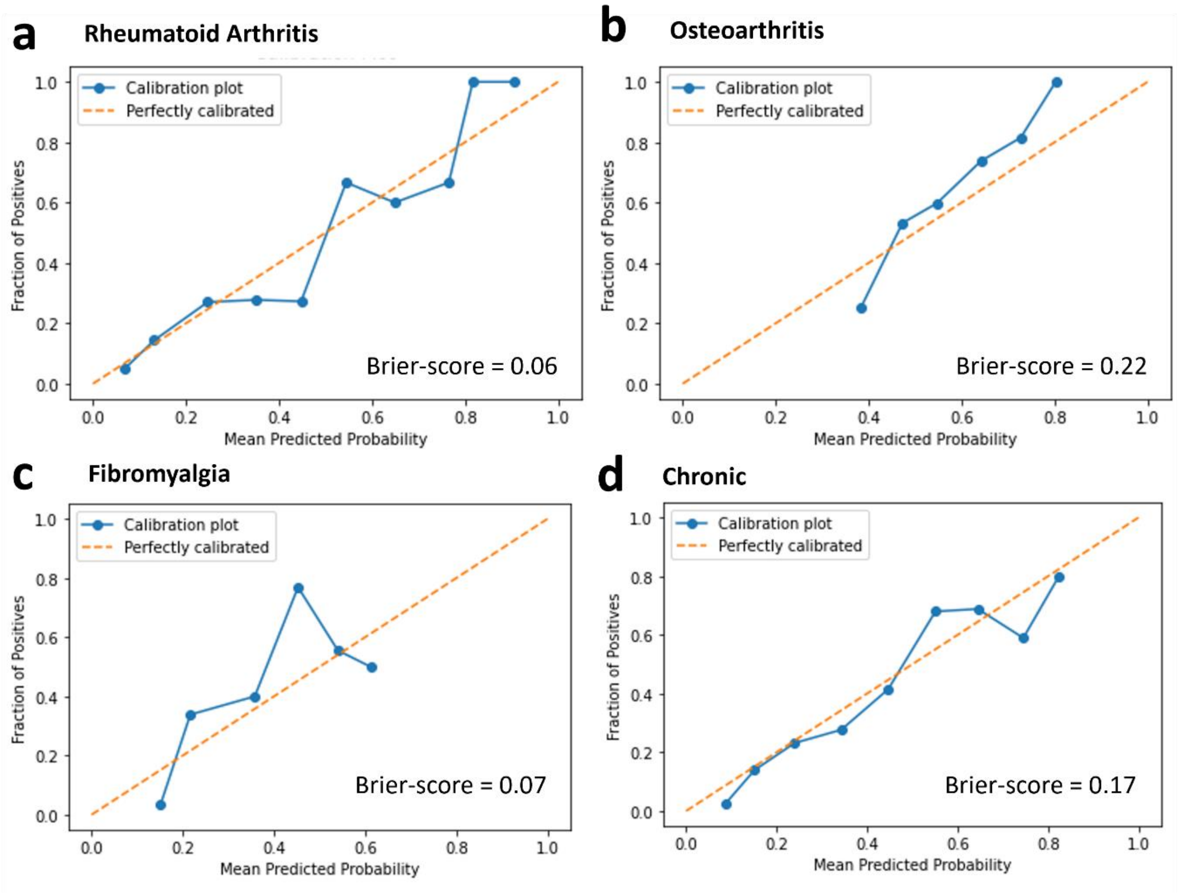

Supplementary Figure 3: Calibration curves showing the agreement between the predicted probabilities and the true likelihood of the patients having the diagnosis, as well as the Brier score. *We show the calibration curves for the four classification tasks:* ***a*** *Rheumatoid arthritis* ***b*** *Osteoarthritis* ***c*** *Fibromyalgia* ***d*** *patients who remain under care of a rheumatologist for >3 months*

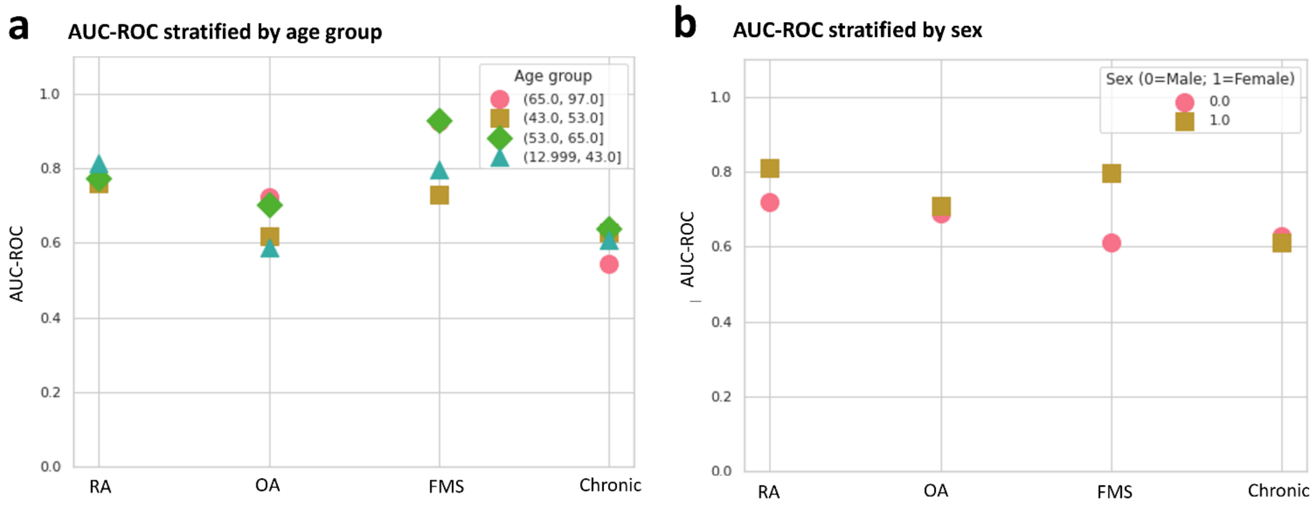

Supplementary Figure 4: AUC-ROC in the validation set for the different classification tasks stratified for age and sex. *Results showing the AUC-ROC performance for the classification of Rheumatoid arthritis (RA), Osteoarthritis (OA), Fibromyalgia and patients who remain under rheumatologist care for more than three months (chronic) across different demographics.* ***a*** *performance in AUC-ROC across age groups* ***b*** *performance stratified for biological sex.*

a Rheumatoid Arthritis

Sens	Spec	PPV	NPV	Acc
0.04	1.00	1.00	0.92	0.92
0.08	1.00	0.67	0.92	0.92
0.15	0.99	0.52	0.93	0.92
0.28	0.96	0.36	0.94	0.90
0.38	0.92	0.29	0.94	0.88
0.71	0.67	0.16	0.99	0.67

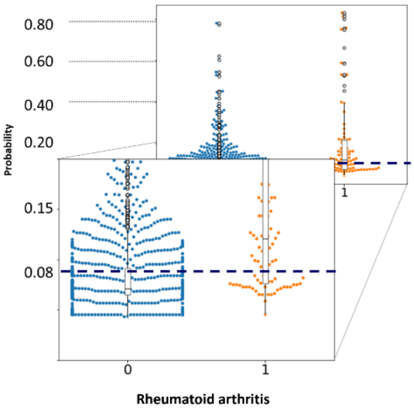

b Osteoarthritis

Sens	Spec	PPV	NPV	Acc
0.04	0.99	0.62	0.79	0.79
0.10	0.99	0.70	0.80	0.80
0.19	0.97	0.63	0.82	0.81
0.24	0.94	0.53	0.82	0.79
0.33	0.92	0.53	0.84	0.80
0.46	0.87	0.48	0.86	0.78

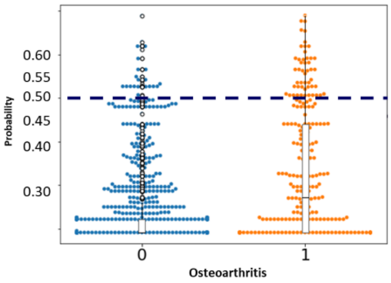

c Chronic

Sens	Spec	PPV	NPV	Acc
0.12	0.95	0.74	0.48	0.51
0.26	0.88	0.71	0.51	0.55
0.47	0.73	0.67	0.54	0.59
0.68	0.50	0.61	0.57	0.59
0.96	0.08	0.55	0.60	0.55
1.00	0.01	0.54	0.57	0.54

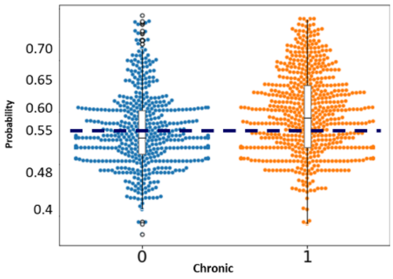

Supplementary Figure 5: Swarm plot showing distribution of cases (=orange) and non-cases (=blue) across a full range of model probabilities and their respective characteristics, to substantiate the choice for the cut-off (=dashed line). *Here we show the swarm plots indicating the model score (=probability) per referral letter (=dot) for the three different classification tasks:* ***a*** *Rheumatoid arthritis* ***b*** *Osteoarthritis* ***c*** *Fibromyalgia* ***d*** *patients who remain under care of a rheumatologist for >3 months.*

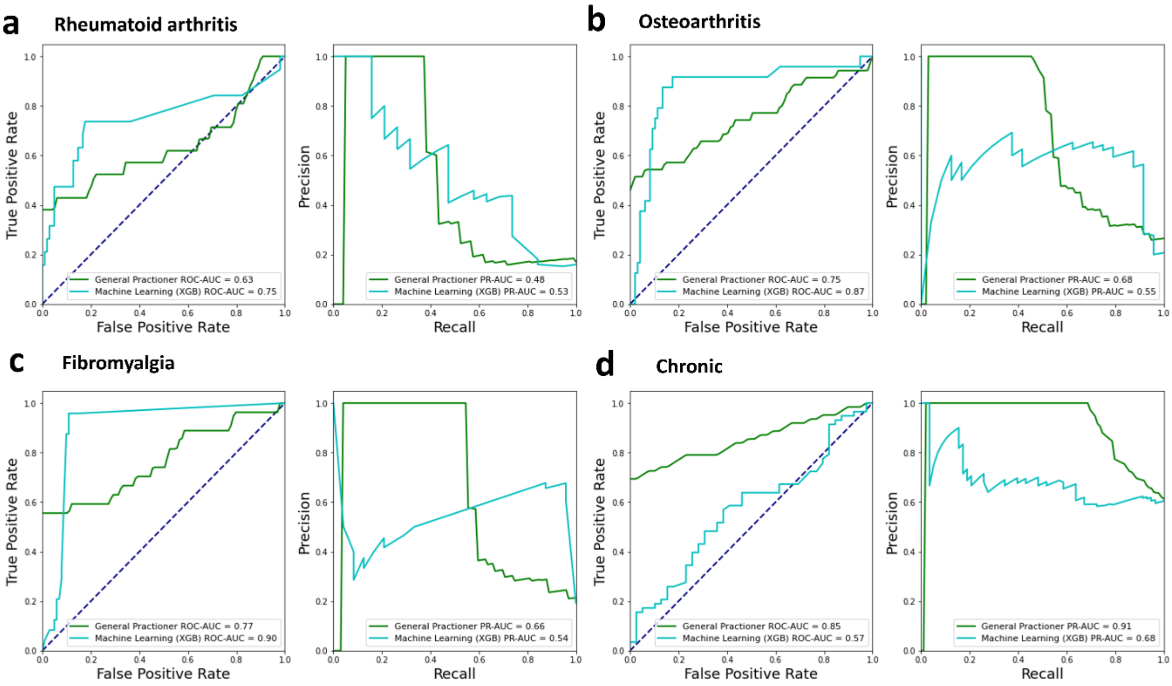

Supplementary Figure 6. Overview of ROC and PR curves of the machine vs the GP's diagnosis for the correct classification (according to a rheumatologist). *We show the ROC- and precision recall (PR) curves for the four classification tasks:* ***a*** *Rheumatoid arthritis* ***b*** *Osteoarthritis* ***c*** *Fibromyalgia* ***d*** *patients who remain under care of a rheumatologist for >3 months. The diagnostic ability of the model is shown in light blue, whereas the ability of GP is shown in green.*

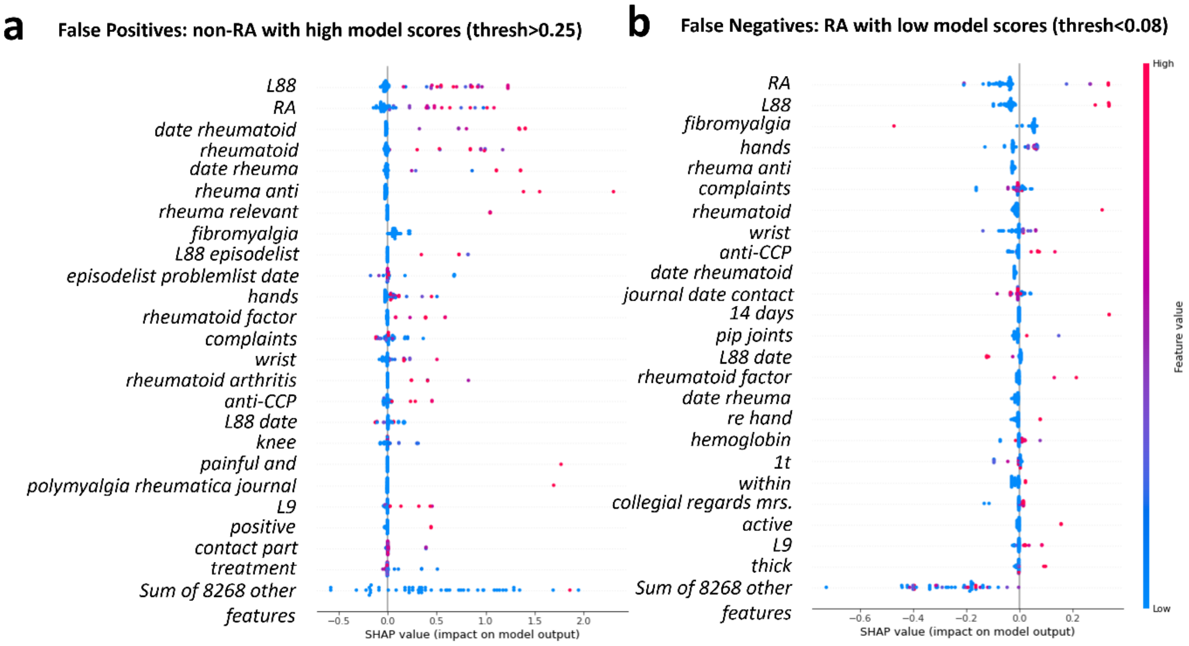

Supplementary Figure 7: Most important keywords driving the false positives & false negative findings. *We applied a SHAP analysis to identify the key terms in the referral letter responsible for either a high or low model score.* ***a*** *keywords that caused the model to assign a high score to non-RA cases, resulting in false positives.* ***b*** *keywords that caused the model to assign a low score to actual RA cases, resulting in false negatives.*

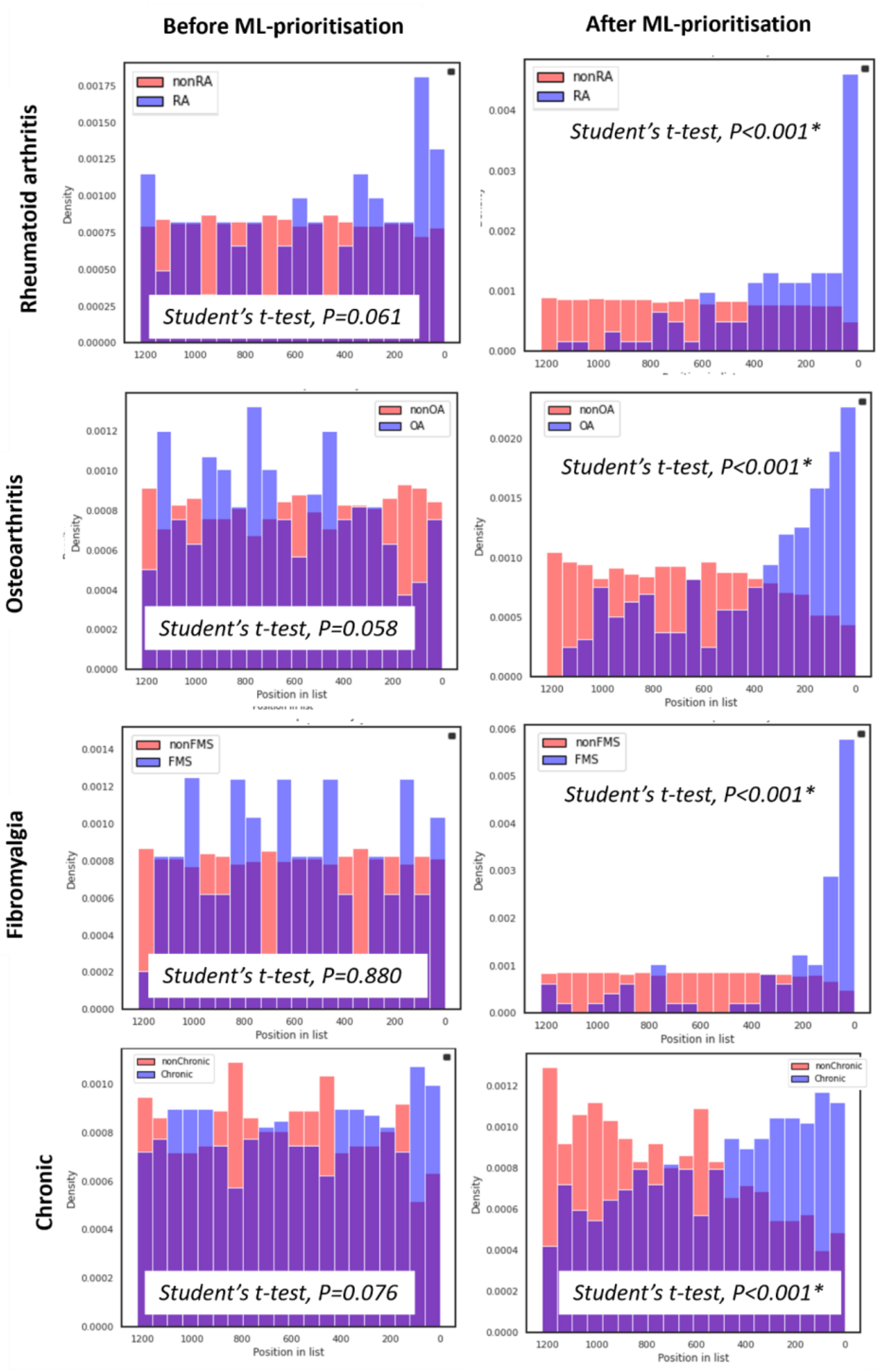

Supplementary Figure 8: The current order of appointments vs the prioritisation according to a ML-technique. *Here, we quantified the discernment in referral order between cases and non-cases based on the Student's t-test.*

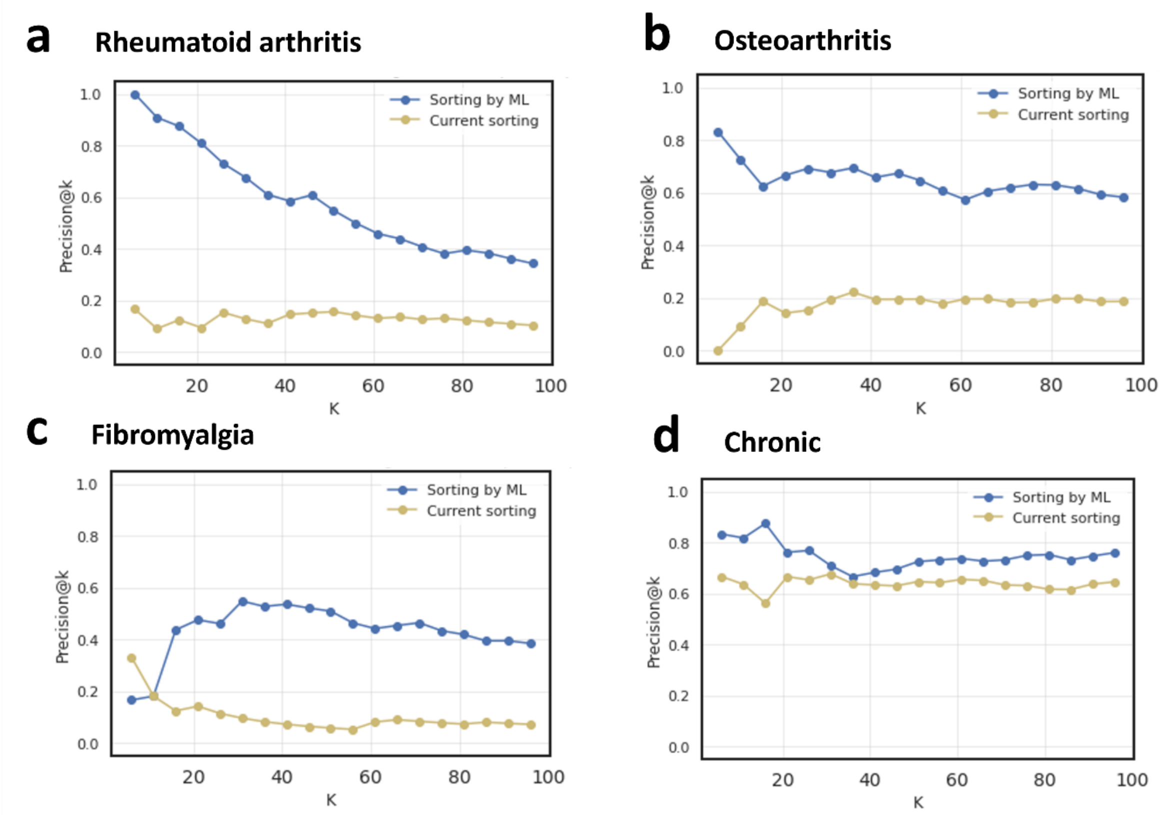

Supplementary Figure 9: The precision of the top K recommendations for different prioritisation techniques versus the current order of appointments. *Here, we compare the ratio of actual cases in the top recommendations between the ML-based prioritisation and the present situation for the four classification tasks:* ***a*** *Rheumatoid arthritis* ***b*** *Osteoarthritis* ***c*** *Fibromyalgia* ***d*** *patients who remain under care of a rheumatologist for >3 months.*

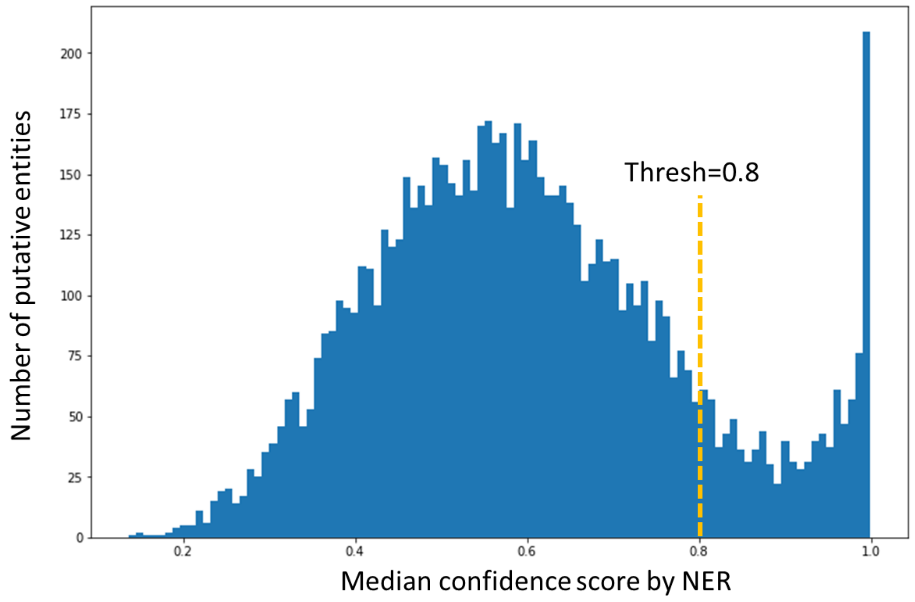

Supplementary Figure 10: Median confidence score for every putative entity captured by the NER-Dutch sequence tagger on the BERTje embedded text. *The cut-off value used to define the entities is indicated by the dashed line.*
